# Global Estimates of the Prevalence of Depression among Prisoners: A Systematic Review and Meta-analysis

**DOI:** 10.1155/2020/3695209

**Published:** 2020-11-26

**Authors:** Asres Bedaso, Mohammed Ayalew, Nibretie Mekonnen, Bereket Duko

**Affiliations:** ^1^College of Medicine and Health Sciences, Faculty of Health, School of Nursing, Hawassa University, Hawassa, Ethiopia; ^2^Australian Centre for Public and Population Health Research, School of Public Health, Faculty of Health, University of Technology Sydney, Ultimo, NSW, Australia; ^3^School of Public Health, Curtin University, Western Australia, Australia

## Abstract

**Background:**

Prison populations tend to be marginalized and disadvantaged of the rights and freedoms that other people in the community benefit from. Their separation from families, a narrow room and lack of privacy in the prison, violence between prisoners, and the uncertainty about the future result in psychological distress, for example, depression. The review has synthesized previous studies conducted on the topic and summarized to formulate recommendations for future prison health care services.

**Methods:**

We systematically searched the databases: PubMed, Psych Info, and SCOPUS, as well as manual Google Scholar searches, were conducted to retrieve published literature globally. We have included observational studies, written in English language. Estimates were pooled using a random-effects model. The study protocol was registered in PROSPERO with protocol number CRD42020156108. Subgroup and sensitivity analysis was conducted, and heterogeneity across the studies was evaluated using Q and the *I*^2^-test. Publication bias was assessed by inspection of the funnel plot and Egger's regression test.

**Result:**

A total of 1313 studies were initially identified through the electronic database; among these, a total of 73 full-text articles were retrieved for further appraisal. Further, 32 full-text articles were included in the final systematic review and meta-analysis. In this meta-analysis, the pooled prevalence of depression among prisoners was 36.9% (95% CI; 27.3-47.6). The pooled prevalence of depression among prisoners in the developing and developed countries was 39.2% and 33.1%, respectively. Moreover, the prevalence of depression was 19.1% and 54% for the studies that used diagnostic and screen tools to diagnose or screen depression, respectively. A leave-one-out analysis revealed that the pooled prevalence of depression among prisoners was not dependent on a single study removal or addition. Thus, the pooled prevalence of depression ranges between 35.3 and 38.0%.

**Conclusion:**

The prevalence of depression among prisoners was high. Therefore, regular and continuous screening of depressive symptoms for prisoners along with its appropriate management is highly recommended.

## 1. Background

Depression is a major psychiatric problem mainly explained by a feeling of depressed mood, sadness, and a loss of interest in activities that you usually enjoy, accompanied by impairment of social and occupational activities for at least two weeks [1]. Depression has a significant contribution to the global burden of disease and affects all communities across the world [1, 2]. The World Health Organization mental health survey conducted across 17 countries found that on average, 1 in 20 people reported having an episode of depression [2].

Different studies conducted among prisoners in several countries have shown a high prevalence of psychiatric morbidity. The prevalence of severe mental disorders among prisoners can be 5 to 10 times higher than in the general population [3]. Depression is especially prevalent in prison population [4]. The overall prevalence of depression among prisoners is found to be 45.5% [5] and 56.4% [6] in Ethiopia, 19.2% [7] in Norway, 57.4% [8] in Turkey, and 72.6% [9] in Nigeria. Unfortunately, offenders with severe mental disorders are ignored by prison officers and staff [10].

Globally, there are more than 10.2 million individuals detained in prisons [11]. Among these, the USA has the highest prison population, around 2.24 million [12]. The rate of prisoners has increased from 136 to 144 per 100,000 of the world population, with a significant difference among the regions. In the last 15 years, the prison population has risen in Latin America with a higher (150%) increment in Brazil [13, 14]. Although it varies among the countries, the prison population has also risen in Asia, Europe, Oceania, and the Gulf region [14]. In Africa, a 76% rise has been observed in Algeria between 2001 and 2003 [14]. While South Africa's prison population rate has decreased from 394 to 328 per 100,000 of the total country's population between 2000 and 2010 [15], Ethiopia's prison population rate has increased from 94 to 124 per 100,000 of the total population between 2000-2011 [13, 14, 16].

Different studies suggest that prisoners are at higher risk of developing mental illness. This can be attributed to many probable factors like incarceration results in loss of personal freedoms and opportunities, such as social supports, interpersonal relationships, employment, social status, and social roles [9]. The condition of a prison such as a narrow room, lack of privacy, violence between prisoners, social neglect, lack of mental health access, and the effects of the prison sentence may lead to depression [15]. In addition, risk factors of depression among prisoners include a family history of a condition, major life changes, chronic health problems, and substance abuse [17].

We found a significant variation in the prevalence of depression among prisoners across the studies conducted globally. However, there are no previous systematic reviews and meta-analysis conducted on the topic of interest. It is hypothesized that a substantial proportion of prisoners would have depression globally. Therefore, this review is aimed at conducting a systematic review and meta-analysis to systematically summarize the magnitude of depression among prisoners and formulate recommendations for future prison health care services.

## 2. Methods

### 2.1. Literature Search Strategy and Selection Process

Preferred reporting items for systematic review and meta-analysis (PRISMA) guideline [18] were used to systematically review the literature (see supplementary file 1). The electronic databases such as PubMed, Scopus, Psych Info, and manual Google Scholar search were searched for relevant articles. A search strategy was developed for each database by using a combination of free texts and Mesh terms. Search terms and the keyword which were used for PubMed are ((“Epidemiology” OR “Prevalence” OR “Magnitude” OR “Incidence”) AND ((“Depression” [Mesh] OR “Depressive Disorder” [Mesh] OR “Major depressive disorder” [Mesh] OR “depressive symptoms” [Mesh] OR (Depression [Title/Abstract] OR Depressive Disorder [Title/Abstract] OR Major depressive disorder [Title/Abstract] OR depressive symptoms” [Title/Abstract])) AND ((offender [Title/Abstract] OR inmates [Title/Abstract] OR prisoner [Title/Abstract] OR person in custody [Title/Abstract]))). Also, we have manually searched Google scholar and the reference lists of review articles, and retrieved full-text articles were also examined for additional papers that were eligible for this review to identify additional literatures. Search limits used in the databases include studies published in English and the period starting from October 1, 2001, to November 10, 2019. This systematic review and meta-analysis protocol has been registered in PROSPERO (CRD42020156108).

### 2.2. Eligibility Criteria

Eligible studies for this review had to fulfill the following criteria for inclusion:

1. Studies which assessed and reported data on the prevalence and/or magnitude of depression among prisoners
2. The type of studies should be observational (cross-sectional, nested case-control, or prospective cohort or retrospective study design).
3. The study participants of reported studies should be prisoners

Exclusion Criteria:

1. Duplicate studies, reviews, clinical trials, commentaries, short communications, and letters to editors
2. Studies published other than English were excluded from the review

### 2.3. Methods for Data Extraction and Quality Assessment

Data extraction was done using specific data extraction format prepared in the Microsoft Excel spreadsheet. After deleting duplicates, each investigator read titles and abstracts closely to include all potential articles. Full-text articles were obtained and reviewed for all criteria. We have resolved the disagreement through discussion and consensus with the research team.

The following information was extracted from eligible full-text articles: author's name, year of publication, country, sample size, study design, the instrument used to assess depression, sampling technique, response rate, and prevalence of depression. The quality of the included studies was evaluated using a modified version of the Newcastle-Ottawa Scale (NOS) [19].

### 2.4. Data Synthesis and Analysis

A Comprehensive Meta-Analysis software (CMA) version 3 was used to conduct all analyses. Studies were pooled to calculate pooled prevalence, odds ratios, and 95% CI using a random-effects model [20]. The magnitude of statistical heterogeneity between studies was assessed using *I*^2^-statistics, and values of 25, 50, and 75% were considered to represent low, medium, and high, respectively [21]. Possible publication bias was assessed by inspection of the funnel plot and Egger's regression tests [22, 23].

## 3. Results

### 3.1. Identification of Studies

**Figure 1:**
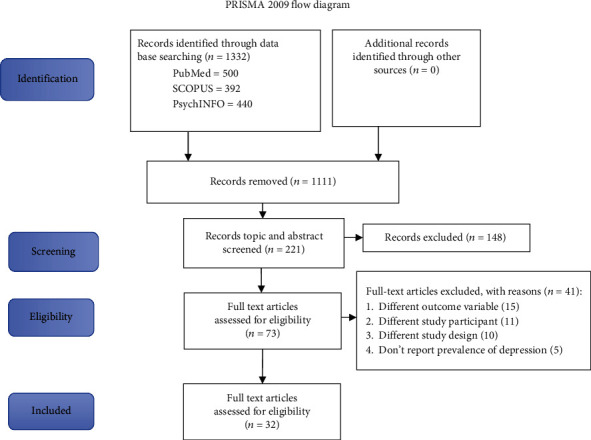
PRISMA flow chart of the study identification process for systematic reviews and meta-analyses, 2019.

The electronic database and additional manual search resulted in a total of 1332 research articles. Of these, a total of 73 full-text articles were retrieved for further appraisal after a careful and organized screening of titles and abstracts. Finally, 32 full-text articles which fulfilled the inclusion criteria were part of the current review (see Figure 1).

### 3.2. Characteristics of Included Studies

We included a total of 32 articles employed in both developed (14 studies) and developing (18 studies) countries representing 101,817 prisoners. The included research articles were published between the year 2001 and 2019. The sample size of the included studies ranges from 24 in the UK to 82,650 in Taiwan. Among the 32 studies, four studies from the USA, three from the UK, three from Brazil, two from Australia, two from France, two from Chile, two from Iran, one from each of the following countries; China, Nepal, Ireland, Norway, Taiwan, and Turkey, and eight from countries in Africa.

Regarding the study design of the included studies, four used a cohort study design, one used case-control, and twenty-seven studies used a cross-sectional study design. Depression among prisoners was assessed using a diagnostic tool such as Composite International Diagnostic Interview (CIDI), Mini-International Neuropsychiatric Interview (MINI), the International Classification of Diseases, 9^th^ version (ICD-9), and the Diagnostic and Statistical Manual of Mental Disorders, fourth edition (DSM-IV) in fourteen studies while a screening tool such as Depression Anxiety Stress Scale (DASS), Patient health question-9 item (PHQ-9), Beck Depression Inventory (BDI), General Health Questionnaire (GHQ-12), Hospital Anxiety and Depression Scale (HADS), Center for Epidemiologic Studies Depression scale (CESD), and Montgomery Asberg Depression Rating Scale (MADRS) in eighteen studies (see Table 1).

### 3.3. Methodological Quality of Included Studies

We have used the modified Newcastle Ottawa Scale (NOS) [19] to assess the methodologic quality of the studies included in the current review. Of the 32 studies included in the analysis, fourteen studies were of high methodologic quality (NOS score ≥ 8), seventeen studies were of moderate methodologic quality (NOS score 6-7), and two studies were low-quality (NOS score ≤ 5) studies (see supplementary file 2).

### 3.4. The Prevalence of Depression among Prisoners (Meta-analysis)

**Figure 2:**
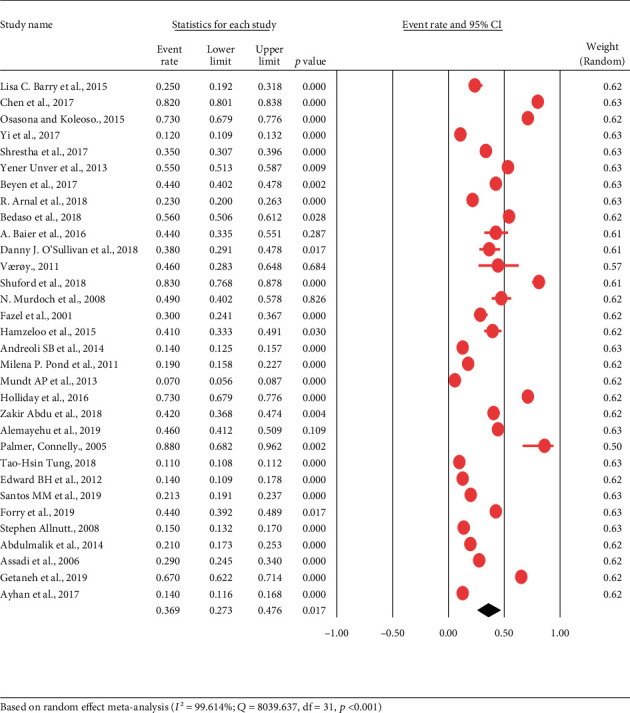
Forest plot for the prevalence of depression among prisoners : a meta-analysis.

In the current review, all analyses were conducted using a Comprehensive Meta-Analysis software (CMA) version 3 [49]. The pooled prevalence estimate of depression among prisoners was 36.9% (95% CI; 27.3-47.6). Because of apparent heterogeneity (*I*^2^ = 99.614%, *Q* = 8039.637, df = 31, *p* < 0.001) identified among the included studies, we employed a random-effects model (see Figure 2).

### 3.5. Subgroup and Sensitive Analysis

We have conducted a subgroup analysis based on the study design (cross-sectional, cohort, and case-control), depression assessment tools used in the study (diagnostic and screening tools), and the place or country in which the study conducted (developed and developing countries). The pooled prevalence of depression among prisoners was higher in developing countries 39.2% (95% CI 27.6-52.2) when compared to developed countries 33.1% (95% CI 23.7-44.0). The pooled prevalence of depression among prisoners was significantly higher in a study used a case-control study design (88.0%) as compared to the cohort (45.6%) and cross-sectional (33.9%) studies. Further, we have used the depression assessment tool to conduct a subgroup analysis. The pooled prevalence of depression was significantly higher (54.0%) among the studies which used screening tools to assess depression whereas remarkably lower among the studies which used a diagnostic tool (19.1%). The heterogeneity was significant for the studies used both diagnostic and screening tools to assess depression, *I*^2^ = 98.407, *p* < 0.0001 and *I*^2^ = 98.220, *p* < 0.001, respectively (see Table 2).

Furthermore, we explored the source heterogeneity through a one-leave-out sensitivity analysis. This analysis revealed that the pooled prevalence of depression among prisoners was not dependent on a single study removal or addition. Thus, the pooled prevalence of depression ranges between 35.3 and 38.0% (see Table 3).

### 3.6. Publication Bias

**Figure 3:**
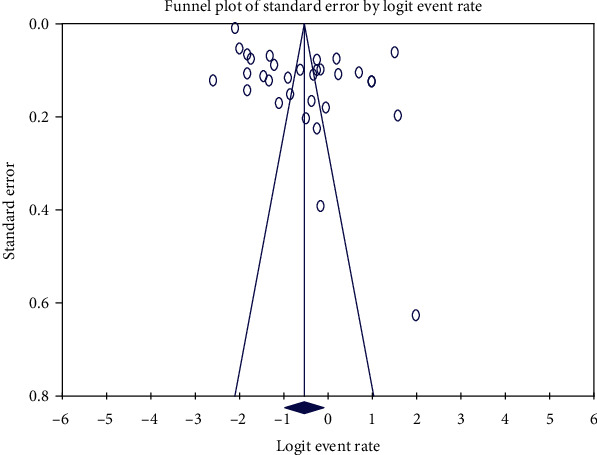
Funnel plot for publication bias of the included studies.

The funnel plot was symmetric, and Egger's regression tests showed no evidence of potential publication bias in the included studies (*B* = 13.87, SE = 2.67, df = 30, *p* = 0.12) (see Figure 3).

## 4. Discussion

In this systematic review and meta-analysis, we explored the prevalence of depression among prisoners. Twenty-seven cross-sectional, four cohort studies, and one nested case-control studies were included in the final meta-analysis. Based on the meta-analysis result, 4 out of 10 prisoners had depression. This implies that depression is a major public health problem among prisoners globally.

In the current study, the overall pooled prevalence estimate of depression among prisoners was 36.9% (95% CI; 27.3-47.6). In our subgroup analysis, the pooled prevalence of depression among prisoners was 39.2% (95% CI 27.6-52.2) in the developing countries and 33.1% (95% CI 23.7-44.0) in developed countries. The reason for this difference might be due to socioeconomic and cultural variations between countries and, also, the utilization of different diagnostic and screening tools for assessing depression, variation in sample size and the difference in the study period.

The result of this meta-analysis was higher than a study conducted on 89037 people from 18 countries concluded that the average lifetime and 12-month prevalence estimates of major depression among the general population were 14.6% and 5.5% in developed countries, whereas 11.1% and 5.9% in developing countries, respectively [52]. Also, the pooled prevalence of depression among developed and developing countries was higher than a study conducted on a community from 30 countries between 1994 and 2014 which revealed a one-year and lifetime prevalence of depression 7.2% and 10.8%, respectively [53]. The possible justification for high prevalence of depression among prisoner might be because of the environment in prison lacks access to social support, poor condition (narrow room, lacks privacy), occurrence of violence between prisoners, limited interpersonal relationship, limited social roles, and lack of mental health access result them in stressful life which might gradually lead them to develop depression [9].

A subgroup analysis of the current review showed the variation in the rates of prevalence of depression among prisoners as the country's economic development differed. The pooled prevalence of depression among prisoners was significantly higher in developing countries (39.2%) when compared to developed countries (33.1%). The possible reason for this variation might be due to economical variation which might lead to a difference in accessing mental health service at the prison level. This finding may be explained by the fact that most of the inmates in a developing country were of low socioeconomic status and may be challenged in accessing health services by themselves [2].

We have also conducted a subgroup analysis using the methodologic design of the studies used as a moderator. The pooled prevalence of depression among prisoners was significantly higher in a study used a nested case-control study design (88.0%) as compared to the cohort (45.6%) and cross-sectional (33.9%) studies. The possible explanations may be we have got only one case-control study, which might overestimate the prevalence when compared with the pooled prevalence of 27 cross-sectional studies. Also, some of the subjects might be deliberately chosen because they have the disease in case-control studies (i.e., that is a higher percentage of cases per study).

We have used the depression assessment tool to conduct a subgroup analysis. The pooled prevalence of depression was significantly higher (54.0%) in the studies using screening tools to assess depression whereas remarkably lower in the studies using a diagnostic tool (19.1%). The possible explanation for the remarkable difference might be due to the variation in measuring instrument (screening and diagnostic) and might be due to difference in the test's ability to correctly identify a participant with the disease as positive and the test's ability to correctly label a participant without the disease as negative [54].

### 4.1. Limitations

The following are limitations of our review which needs to be considered when interpreting our findings. First, only studies published in the past ten years were part of the current review. Second, inclusion of articles only published in English could be another limitation. However, the strength of this study includes screening of articles was conducted by two independent investigators to minimize the possible reviewer bias, and we also conducted a subgroup, and one-leave-out sensitivity analysis to further know source of heterogeneity among the included studies.

## 5. Conclusion

In our meta-analysis, the pooled prevalence of depression among prisoners was high (36.9%). The pooled prevalence of depression was 54.0% and 19.1% among studies which used screening and diagnostic tool, respectively. Attention needs to be given for the mental health of the prisoners, and work should be done to consider the possible integration of mental health services with the existing health care at the prison level. Finally, future studies need to be conducted using a validated instrument to be used in the prison community and to better assess determinants of depression among prisoners as well.

## Figures and Tables

**Table 1:** Characteristics of included studies on the prevalence of depression among prisoners.

Author, publication year	Study design	Sample size	Measurement	Prevalence (%) (95% CI)	Response rate (%)	Sampling technique	Country
Lisa C. Barry et al., 2015 [24]	Cross-sectional	167 (M = 110, F = 57)	PHQ-9	25 (19.2-31.8)	92.70%	Simple RS	USA
Chen et al., 2017 [25]	Cross-sectional	1705 (M = 1509, F = 646)	BDI	81.5 (80.1-83.8)	96.20%	Multistage	China
Osasona and Koleoso, 2015 [9]	Cross-sectional	323 (M = 229, F = 23)	HADS	72.60 (67.9-77.6)	72.42%	Systematic RS	Nigeria
Yi et al., 2017 [26]	Longitudinal survey	3,139 (M = 3139, F = 0)	CIDI	11.60 (10.9-13.2)	98%	Purposive ST	USA
Shrestha et al., 2017 [27]	Cross-sectional	434 (M = 434, F = 0)	CESD	35.3 (30.7-39.6)	96.60%	Simple RS	Nepal
Yener Unver et al., 2013 [8]	Cross-sectional	685 (M = 685, F = 0)	DASS-42	54.70 (51.3-58.7)	100%	Convenience	Turkey
Beyen et al., 2017 [28]	Cross-sectional	649 (M = 583, F = 66)	PHQ-9	43.80 (40.2-47.8)	89.20%	Multistage	Ethiopia
Arnal R. et al., 2018 [29]	Comparative CS	694 (M = 694, F = 0)	DSM-IV	22.80 (20.0-26.3)	99.80%	Stratified	France
Bedaso et al., 2018 [6]	Cross-sectional	335 (M = 327, F = 8)	PHQ-9	56.40 (50.6-61.2)	100%	Simple RS	Ethiopia
Baier A. et al., 2016 [30]	Prospective cohort	79 (M = 51, F = 28)	MINI	44 (33.5-55.1)	99%	Simple RS	Chile
Danny J. O'Sullivan et al., 2018 [31]	Cross-sectional	101 (M = 101, F = 0)	BDI-II	37.60 (29.1-47.8)	100%	Convenience	Ireland
Værøy, 2011 [7]	Cross-sectional	26 (M = 26, F = 0)	MADRS	46.10 (2.3-64.8)	100%	Simple RS	Norway
Shuford et al., 2018 [32]	Longitudinal study	179 (M = 0, F = 179)	CESD-10	83 (76.8-87.8)	100%	Simple RS	USA
Murdoch N. et al., 2008 [33]	Population-based survey	121 (M = 121, F = 0)	Geriatric Depression Scale	49 (40.2-57.8)	99%	All participants	UK
Fazel et al., 2001 [34]	Cross-sectional	203 (M = 203, F = 0)	DSM IV	29.60 (24.1-36.7)	87.10%	Stratified	England
Hamzeloo et al., 2015 [35]	Cross-sectional	147 (M = 147, F = 0)	BDI-II	40.80 (33.3-49.1)	100%	Stratified	Iran
Andreoli SB et al., 2014 [36]	Cross-sectional	1809 (M = 1809, F = 0)	CIDI	14.20 (12.5-15.7)	100%	Stratified	Brazil
Milena P. Pond et al., 2011 [37]	Cross-sectional	497 (M = 497, F = 0)	MINI(Portuguese version)	18.80 (15.8-22.7)	100%	Simple RS	Brazil
Mundt AP et al., 2013 [38]	Cross-sectional	1008 (M = 855, F = 153)	CIDI	6.90 (5.6-8.7)	100%	Simple RS	Chile
Holliday et al., 2016 [39]	Cross-sectional	320 (M = 320, F = 0)	PHQ-9	72.80 (67.9-77.6)	100%	Convenience	USA
Zakir Abdu et al., 2018 [40]	Cross-sectional	332 (M = 311, F = 21)	BDI-II	41.90 (36.8-47.4)	99%	Systematic RS	Ethiopia
Alemayehu et al., 2019 [5]	Cross-sectional	402 (M = 394, F = 8)	PHQ-9	45.50 (41.2-50.9)	95.20%	Simple RS	Ethiopia
Palmer, Connelly, 2005 [41]	Case control	24 (M = 24, F = 0)	BDI-II	87.50 (68.2-96.2)	100%	Purposive	UK
Tao-Hsin Tung, 2018 [42]	Survey	82,650 (M = 74,130, F = 8520)	ICD-9	11.31 (10.8-11.2)	100%	Purposive	Taiwan
Edward BH et al., 2012 [42]	Cross-sectional	396 (M = 347, F = 72)	CIDI	14.30 (10.9-17.8)	100%	Systematic RS	Australia
Santos MM et al., 2019 [43]	Cross-sectional	1809 (M = 1192, F = 617)	CIDI	21.30 (19.1-23.7)	90%	Multistage	Brazil
Forry et al., 2019 [44]	Cross-sectional	414 (M = 397, F = 17)	M.I.N.I-6.0	44 (39.2-48.9)	95.60%	Simple RS	Uganda
Stephen Allnutt, 2008 [45]	Cross-sectional	1322 (M = 1104, F = 218)	CIDI	15 (13.2-17.0)	100%	Simple RST	Australia
Abdulmalik et al., 2014 [46]	Cross-sectional	394 (M = 389, F = 5)	GHQ-12	20.80 (17.3-25.3)	100%	Multistage	Nigeria
Assadi et al., 2006 [51]	Cross-sectional	351 (M = 351, F = 0)	DSM-IV	29 (24.5-34.0)	100%	Stratified RS	Iran
Getaneh et al., 2019 [47]	Cross-sectional	400 (M = 378, F = 22)	PHQ-9	66.50 (62.1-71.4)	94.80%	Simple RS	Ethiopia
Ayhan et al., 2017 [48]	Cross-sectional	707 (M = 647, F = 60)	MINI	14.30 (11.6-16.8)	100%	Systematic RS	French

**Table 2:** Subgroup analysis for the meta-analysis of the prevalence of depression among prisoners.

Subgroups	No. of studies	Prevalence (%)	95% CI	Heterogeneity within the study *(I^2^ and Q)*		Heterogeneity between groups (*p* value)
				*Q* value	*I* ^2^ (%)	
Study design						
Cohort	4	45.6	12.8-82.8	415.472	92.278	<0.001
Case-control	1	88.0	68.2-96.2	0.00	0.00	
Cross-sectional	27	33.9	23.8-45.8	7575.659	99.657	
Country						
Developed	14	33.1	23.7-44.0	1366.316	99.049	<0.001
Developing	18	39.2	27.6-52.2	2339.710	99.273	
Tools used to assess depression						
Diagnostic tool	14	19.1	15.0-23.9	815.867	98.407	<0.001
Screening tool	19	54.0	43.8-63.9	954.901	98.220	

**Table 3:** One-leave-out sensitivity analysis of the prevalence of depression among prisoners for each study being removed at a time (prevalence and 95% confidence).

Study excluded	Prevalence (%)	95% CI
Lisa C. Barry et al., 2017 [24]	37.3	27.5-48.3
Chen et al., 2017 [25]	35.2	27.0-44.3
Osasona and Koleoso, 2015 [9]	35.7	26.4-46.3
Yi et al., 2017 [26]	38.0	27.7-49.6
Shrestha et al., 2017 [27]	36.9	27.2-47.9
Yener Unver et al., 2013 [8]	36.3	26.8-47.0
Beyen et al., 2017 [28]	36.7	27.0-47.5
Arnal R., et al., 2018 [29]	37.4	27.5-48.5
Bedaso et al., 2018 [6]	36.3	26.7-47.0
Baier A., et al., 2016 [30]	36.7	27.0-47.5
Danny J. O'Sullivan et al., 2018 [31]	36.8	27.1-47.7
Værøy, 2011 [7]	36.6	27.0-47.5
Sheford et al., 2018 [32]	35.3	27.1-47.7
Murdoch N., et al., 2008 [33]	36.5	27.0-47.5
Fazel et al., 2001 [34]	37.1	26.0-45.9
Hamzeloo et al., 2016 [35]	36.7	26.9-47.4
Andreoli SB et al., 2014 [36]	37.9	27.3-48.1
Milena P. Pond et al., 2011 [37]	37.6	27.7-48.6
Mundt AP et al., 2013 [38]	38.4	28.5-49.5
Holliday et al., 2016 [39]	35.7	26.4-46.3
Zakir Abdu et al., 2018 [40]	36.6	26.9-47.5
Alemayehu et al., 2019 [5]	36.7	27.0-47.6
Palmer, Connelly, 2005 [41]	35.3	25.9-46.1
Tao-Hsin Tung, 2019 [50]	38.0	28.8-48.2
Edward BH et al., 2012 [42]	37.8	27.9-48.9
Santos MM et al., 2019 [43]	37.5	27.4-48.7
Forry et al., 2019 [44]	36.7	27.0-47.5
Stephen Allnutt, 2008 [45]	37.8	27.7-49.1
Abdul Malik et al., 2006 [46]	37.5	27.6-48.5
Assadi et al., 2006 [51]	37.2	27.3-48.1
Getaneh et al., 2019 [47]	35.9	26.5-46.5
Ayhan et al., 2017 [48]	37.9	27.9-49.0

## Abbreviations

BDI: Beck Depression Inventory
CESD: Centre for Epidemiologic Studies Depression scale
CI: Confidence interval
CIDI: Composite International Diagnostic Interview (CIDI)
DASS: Depression Anxiety Stress Scale
DSM-IV in fourteen studies while a screening tool such as DSM-IV: A diagnostic and statistical manual of mental disorders, 4th edition
GHQ-12: General Health Questionnaire
HADS: Hospital Anxiety and Depression Scale
ICD-9: The International Classification of Diseases, 9th version (ICD-9)
MADRS: Montgomery Asberg Depression Rating Scale
MIN: Mini-International Neuropsychiatric Interview (MINI; PHQ-9: Patient health question-9 item)
PRISMA: Preferred Reporting Items for Systematic Reviews and Meta-Analyses
RS: Random sampling
RST: Random sampling techniques.

## Supplementary Materials

Supplementary Materials Supplementary file 1: PRISMA 2009 Checklist. Supplementary file 2: summary of the quality score and agreed level of bias and level of agreement on the methodological qualities of included studies in a meta-analysis based on sampling, outcome, response rate, and method of analysis.
